# Effects of tillage and straw return on water-stable aggregates, carbon stabilization and crop yield in an estuarine alluvial soil

**DOI:** 10.1038/s41598-019-40908-9

**Published:** 2019-03-14

**Authors:** Ke Song, Xianqing Zheng, Weiguang Lv, Qin Qin, Lijuan Sun, Hanlin Zhang, Yong Xue

**Affiliations:** 1 Institute of Eco-Environmental and Plant Protection, Shanghai Academy of Agricultural Sciences, Shanghai, 201403 China; 2 Scientific Observation Experimental Station, Shanghai Agricultural Environment and Cultivated Land Conservation, Ministry of Agriculture, Shanghai, 201403 China; 3 Shanghai Research Center of Low-carbon Agricultural Engineering Technology, Shanghai, 201403 China

## Abstract

In China, the average soil organic carbon (SOC) content of cultivated land is 30% less than the world average. Therefore, cultivation management-induced changes in SOC dynamics are necessary, especially in estuarine alluvial islands, where the SOC stocks are limited. We studied the effect of different combinations of tillage, fertilization and straw return on C distribution in different soil aggregates and on crop yield on an estuarine alluvial soil in eastern China. Compared to conventional tillage, conservation tillage (no-tillage coupled with straw return) increased water-stable large macroaggregates (>2 mm) by 35.18%, small macroaggregates (2–0.25 mm) by 33.52% and microaggregates by 25.10% in the topsoil (0–20 cm). The subsoil (20–40 cm) also showed the same trend. Compared to conventional tillage without straw return, large and, small macroaggregates and microaggregates in conservation tillage were increased by 24.52%, 28.48% and 18.12%, respectively. Straw return also caused a significant increase in aggregate-associated carbon (aggregate-associated C). No-tillage coupled with straw return had more total aggregate-associated C within all the aggregate fractions in the topsoil. But the different is that conventional tillage with straw return resulted in more aggregate-associated C than conservation tillage in the subsoil. No-tillage combined with straw return (T8) produced the highest carbon preservation capacity (CPC) of macroaggregates and microaggregates in the topsoil. A considerable proportion of the SOC was found to be stocked in the small macroaggregates under both topsoil (74.56%) and subsoil (67.09%). The CPC was highest (19.17 g·kg^−1^) in small macroaggregates. However, no-tillage and straw return had less potential to sustain crop yield than did the conventional tillage practices; with the average rice and wheat yield correspondingly decreased by 10.63% and 7.82% in three years.

## Introduction

Soil organic carbon (SOC) is an important soil component that plays a crucial role in soil fertility^1^, environmental protection^2^ and sustainable agricultural development^3^. It has therefore been regarded as the foundation of soil quality and function^1^. Farmland SOC sequestration is closely related to the reduction of CO_2_ emissions^4^, the enhancement of soil fertilization, the maintenance of soil structure^5^, and the promotion of microbial diversity^6,7^, among other items. Hence, it is the decisive factor affecting the quality of cultivated land and crop yield^1,8^. However, the SOC content in Chinese farmland soil is generally low^9^, which is lower than the world average by more than 30% and that of Europe by more than 50%^10^. Therefore, the improvement of the SOC content of cultivated soil has been a topic of great concern in the field of agricultural science. In addition to the influence of natural factors such as regional weather and soil conditions^11,12^, the variation in the agriculture SOC stock is most strongly affected by human activities^2,13^. The effect of management practices on farmland SOC content has been extensively investigated, and most studies have indicated that conservation farming measures (e.g., no-tillage, application of organic fertilizer, and straw return) not only increase the agriculture SOC stock^14–17^, but also improve crop yield^18,19^. These measures mainly increase farmland SOC content by increasing SOC input and improving soil aggregate retention^20–22^. However, some studies have suggested that although no-tillage and straw return are beneficial to SOC accumulation, they may also reduce crop yield^23,24^. Moreover, currently, organic fertilizers have mostly been applied to orchards and vegetable plots that are economically intensive. In contrast, they have rarely been applied to field crops and thus have made little contribution to field carbon inputs^25^. Therefore, these studies raise new questions. Firstly, among various farming management measures, which measure is most effective in retaining SOC, and which aggregate distribution relationship is involved? Secondly, is straw return more effective than organic fertilizer application in retaining SOC, and how can carbon sequestration capacity be quantitatively represented? Thirdly, do no-tillage and straw return truly lower crop yield?

To answer these questions, this study was conducted in estuarine alluvial soil from Chongming Island, Eastern China. Chongming Island is an alluvial island in the Yangtze River Delta. Estuary alluvial soil is the main soil type. Because the estuarine alluvial soil was formed recently and the island is surrounded by vast wetlands and beaches, the soil is usually characterized by high salinity and low soil nutrient and organic matter content. Therefore, it is very important to improve soil structure and increase soil organic carbon content in this region by better agricultural management.

The major agricultural system in this region is rice-wheat cropping rotation. Double cropping is characterized by intensive farming with high application of mineral fertilizers and no organic fertilizer^26^. This leads to a continuous reduction of SOC resulting in a low SOC stock (The average is 9.32 g·kg^−1^). Therefore, the cultivation of rice and wheat crops grown rotationally with different tillage, fertilization and residue return practices was tested to evaluate the long-term effect of these cultivation practices on aggregation, C stabilization and yield attributes in estuarine alluvial soil. We hypothesized that no-tillage with straw return and chemical fertilizers could improve soil aggregation, C sequestration and sustainable yield increment in rice-wheat cropping rotation systems.

## Results

### Different types of soil carbon

The effects on soil carbon of 3 years of rice-wheat cropping rotation are shown in Table 1. The average topsoil contents of total carbon (TC), SOC and labile organic carbon (LOC) were 12.72 g·kg^−1^, 11.01 g·kg^−1^, and 7.13 g·kg^−1^, respectively, under the three no-tillage treatments (T7, T8, and T9). These mean values were significantly higher than those under the conventional tillage treatments (T1, T2 and T3), which had average TC, SOC, and LOC contents of 9.87 g·kg^−1^, 8.56 g·kg^−1^, and 5.20 g·kg^−1^, respectively. The average contents of the three carbon types under rotary tillage were between those under conventional tillage and those under no-tillage. The contents of TC and SOC were significantly lower than those under no-tillage. The subsoil contents of TC, SOC, and LOC under conventional tillage were 9.42 g·kg^−1^, 7.60 g·kg^−1^, and 5.97 g·kg^−1^, respectively, which were 17.16%, 4.25% and 16.83% higher than those under no-tillage.

**Table 1:** Soil total carbon (TC), soil organic carbon (SOC) and soil labile organic carbon (LOC) in topsoil and subsoil under different treatments after 3 years of rice-wheat cropping rotation.

Treatments	TC (g·kg^−1^)		SOC (g·kg^−1^)		LOC (g·kg^−1^)	
	0–20 cm	20–40 cm	0–20 cm	20–40 cm	0–20 cm	20–40 cm
T1	8.74 ± 0.07d	8.34 ± 0.06bc	7.88 ± 0.66d	5.59 ± 0.05b	4.67 ± 0.69c	5.47 ± 0.07bc
T2	10.78 ± 1.00abc	9.97 ± 0.04a	9.06 ± 0.16 cd	8.72 ± 0.06a	5.61 ± 0.13abc	6.81 ± 0.04a
T3	10.11 ± 0.43bc	9.94 ± 0.12a	8.73 ± 0.36 cd	8.48 ± 0.14a	5.32 ± 0.11bc	5.63 ± 0.17bc
T4	11.89 ± 1.90abc	8.66 ± 0.14ab	9.76 ± 0.07bc	8.04 ± 0.03a	6.05 ± 0.42abc	6.30 ± 0.26ab
T5	10.25 ± 0.37bc	7.76 ± 0.03bc	8.86 ± 0.35 cd	5.88 ± 0.04b	5.53 ± 0.47abc	4.84 ± 0.05c
T6	12.64 ± 0.51abc	9.78 ± 0.05a	11.03 ± 0.22ab	7.94 ± 0.24a	7.04 ± 0.34abc	6.27 ± 0.05ab
T7	9.84 ± 0.32bc	7.15 ± 0.03c	8.95 ± 0.64 cd	6.63 ± 0.03ab	5.70 ± 0.69abc	4.82 ± 0.15c
T8	14.57 ± 0.57a	8.72 ± 0.06ab	12.36 ± 0.12a	8.22 ± 0.02a	8.16 ± 0.73a	5.39 ± 0.16bc
T9	13.75 ± 0.43ab	8.26 ± 0.22bc	11.69 ± 0.03a	8.03 ± 0.12a	7.54 ± 0.73ab	5.11 ± 0.24bc

Mean values are shown ± SEs (n = 3), and different lowercase letters in the same column indicate significantly differences at *P* < *0*.*05*.

T1: Conventional tillage with chemical fertilizer; T2: Conventional tillage with straw return and chemical fertilizer; T3: Conventional tillage with organic fertilizer and chemical fertilizer; T4: Rotary tillage with chemical fertilizer; T5: Rotary tillage with straw return and chemical fertilizer; T6: Rotary tillage with organic fertilizer and chemical fertilizer; T7: No-tillage with chemical fertilizer; T8: No-tillage with straw return and chemical fertilizer; T9: No-tillage with organic fertilizer and chemical fertilizer.

In addition to the influence of tillage, straw return and organic fertilizer also led to variations in soil carbon content among the different treatments. The average topsoil TC contents under straw return (T2, T5 and T8) and organic fertilizer (T3, T6 and T9) were 16.83% and 19.78% higher than those under chemical fertilizer only (T1, T4 and T7), with F = 6.852 and P < 0.05. Similar results were observed for the SOC and LOC. The average subsoil contents of TC, SOC, and LOC under straw return were 8.82 g·kg^−1^, 7.61 g·kg^−1^, and 5.68 g·kg^−1^, respectively. The corresponding values under organic fertilizer were 9.33 g·kg^−1^, 8.15 g·kg^−1^, and 5.67 g·kg^−1^, respectively. The subsoil TC and SOC contents under straw return were significantly higher than those under chemical fertilizer only, but no significant differences were observed in the LOC content.

T1: Conventional tillage with chemical fertilizer; T2: Conventional tillage with straw return and chemical fertilizer; T3: Conventional tillage with organic fertilizer and chemical fertilizer; T4: Rotary tillage with chemical fertilizer; T5: Rotary tillage with straw return and chemical fertilizer; T6: Rotary tillage with organic fertilizer and chemical fertilizer; T7: No-tillage with chemical fertilizer; T8: No-tillage with straw return and chemical fertilizer; T9: No-tillage with organic fertilizer and chemical fertilizer.

### Aggregate size distribution

The relative percentages of soil aggregates obtained by wet sieving are shown in Table 2. The topsoil contents of large macroaggregates (>2 mm), small macroaggregates (2–0.25 mm), and microaggregates (0.25–0.053 mm) were approximately 10%, 50% and 20%, respectively. The subsoil contents of the three size aggregates had similar distribution trends but were lower than those of the topsoil.

**Table 2:** Large macroaggregates (LMa), small macroaggregates (SMa) and microaggregates (Mi) in topsoil and subsoil under different treatments after 3 years of rice-wheat cropping rotation.

Treatments	LMa > 2 mm (%)		SMa 2-0.25 mm (%)		Mi 0.25–0.053 mm (%)	
	0–20 cm	20–40 cm	0–20 cm	20–40 cm	0–20 cm	20–40 cm
T1	7.59 ± 4.28 cd	1.13 ± 0.08 f	50.67 ± 1.36e	33.25 ± 0.84d	18.62 ± 2.12 cd	11.12 ± 0.24d
T2	7.05 ± 2.77d	7.62 ± 0.34ab	48.52 ± 0.85d	36.41 ± 0.29c	16.53 ± 0.04e	14.63 ± 0.48c
T3	5.31 ± 0.45e	3.46 ± 0.64d	56.46 ± 1.10c	37.42 ± 0.85c	18.04 ± 0.54d	15.49 ± 1.18bc
T4	9.81 ± 1.83ab	2.52 ± 1.18e	45.48 ± 0.19de	45.53 ± 1.67bc	22.65 ± 0.19a	12.64 ± 1.82d
T5	8.46 ± 0.74c	6.51 ± 1.39b	62.07 ± 0.46b	49.42 ± 2.57b	18.43 ± 0.46 cd	17.48 ± 1.29b
T6	7.56 ± 2.41 cd	4.68 ± 0.66c	59.87 ± 1.18bc	38.64 ± 2.60c	19.78 ± 1.83c	16.22 ± 1.20b
T7	9.61 ± 1.81b	6.38 ± 1.48b	62.11 ± 1.67b	46.20 ± 0.54bc	20.68 ± 1.42b	14.63 ± 0.40c
T8	10.26 ± 3.69a	8.21 ± 1.74a	67.65 ± 3.20a	56.48 ± 1.18a	19.64 ± 1.19c	19.74 ± 0.66a
T9	9.72 ± 2.77ab	8.16 ± 0.82a	66.87 ± 0.62a	55.77 ± 1.86a	20.53 ± 0.23b	20.35 ± 1.81a
**Mean**	**8**.**36**	**5**.**07**	**58**.**07**	**44**.**32**	**19**.**42**	**15**.**81**

Mean values are shown ± SEs (n = 3), and different lowercase letters in the same column indicate significant differences at *P* < *0*.*05*. Treatment abbreviations are as listed in Table 1.

No-tillage and straw return caused a significant increase in the contents of macroaggregates and microaggregates, especially in the topsoil. As shown in Table 2, no-tillage (T7, T8 and T9) increased the numbers of large macroaggregates (11.25%) and small macroaggregates (9.45%) compared to those under conventional tillage (T1, T2, and T3). A similar trend was observed in the subsoil. Under the same tillage, the order of large and small macroaggregate contents in the topsoil and subsoil was as follows: straw return >organic fertilizer >single application of chemical fertilizer. In particular, more large macroaggregates were observed under no-tillage than under straw return (T8), and these values were 6.76% and 28.68% higher than those under the single application of fertilizer (T7) in the topsoil and subsoil, respectively.

### Organic carbon in soil aggregates

As shown in Table 3, the aggregate-associated C content within varied aggregate sizes was significantly higher in the topsoil than in the subsoil. The order was as follows: small macroaggregates >microaggregates >large macroaggregates, with average values of 25.14 g·kg^−1^, 23.34 g·kg^−1^, and 20.54 g·kg^−1^, respectively. In contrast to the topsoil, the variation in aggregate -associated C in the subsoil was smaller between the different aggregate sizes. The average contents under the different treatments were from 10.42–11.77 g·kg^−1^.

**Table 3:** Large macroaggregate-C (LMa-C), small macroaggregate-C (SMa-C) and microaggregate-C (Mi-C) in topsoil and subsoil under different treatments after 3 years of rice-wheat cropping rotation. Mean values are shown ± SEs (n = 3), and different lowercase letters in the same column indicate significant differences at *P* < *0*.*05*. Treatment abbreviations are as listed in Table 1.

Treatments	LMa-C (g·kg^−1^)		SMa-C (g·kg^−1^)		Mi-C(g·kg^−1^)	
	0–20 cm	20–40 cm	0–20 cm	20–40 cm	0–20 cm	20–40 cm
T1	18.88 ± 0.06d	10.49 ± 0.58b	22.12 ± 0.49d	12.24 ± 0.37ab	21.25 ± 0.80d	12.05 ± 0.23a
T2	21.06 ± 0.92bc	11.64 ± 0.12a	23.46 ± 0.12c	12.51 ± 0.32a	23.17 ± 0.35b	12.18 ± 0.09a
T3	20.39 ± 0.58c	11.91 ± 0.27a	24.76 ± 0.24bc	12.75 ± 0.12a	22.86 ± 1.04c	12.37 ± 0.58a
T4	18.41 ± 0.07d	10.35 ± 0.23b	23.62 ± 0.37c	10.80 ± 0.12bc	22.11 ± 1.84c	11.31 ± 0.12b
T5	20.35 ± 0.28c	10.43 ± 0.23b	25.51 ± 1.10b	11.18 ± 0.11b	23.62 ± 2.32b	12.10 ± 0.18a
T6	20.21 ± 1.09c	10.86 ± 0.35b	25.30 ± 0.86b	11.47 ± 0.12b	23.45 ± 0.35b	11.84 ± 0.58b
T7	20.48 ± 0.80c	9.92 ± 0.46c	25.18 ± 0.73b	10.12 ± 0.80c	23.18 ± 0.12b	11.05 ± 0.57b
T8	23.51 ± 2.47a	11.11 ± 0.12ab	28.42 ± 0.12a	11.46 ± 0.12b	25.10 ± 0.58a	11.46 ± 0.23b
T9	21.86 ± 0.17b	10.10 ± 0.23bc	27.80 ± 1.25a	11.19 ± 0.35ab	25.25 ± 0.46a	11.58 ± 0.23b
Mean	**20**.**54**	**10**.**77**	**25**.**14**	**11**.**51**	**23**.**34**	**11**.**77**

In the topsoil, no-tillage (T7) showed a significantly higher aggregate-associated C than that under conventional tillage (T1) and rotary tillage (T4). There was no significant difference in aggregate-associated C between conventional and rotary tillage. Under the same tillage conditions, the aggregate-associated C contents under straw return and organic fertilizer were higher than those under single chemical fertilizer. No-tillage coupled with straw return (T8) had the highest aggregate-associated C in all treatments. The associated C contents of large and small macroaggregates and microaggregates were 25.04%, 28.55%, and 18.12% higher, respectively, than those under conventional tillage (T1), which had the lowest aggregate-associated C.

In contrast to the topsoil, the aggregate-associated C contents in the subsoil showed the trend of conventional tillage >rotary tillage >no-tillage. Without straw return and organic fertilizer, the average contents of aggregate-associated C were 11.60 g·kg^−1^, 10.83 g·kg^−1^ and 10.33 g·kg^−1^, respectively, under the T1, T4, and T7 treatments. T1 was significantly greater than T4 and T7 (P < 0.05). Under the same tillage, the application of organic fertilizer and straw return increased the content of aggregate-associated C in the subsoil. Under conventional tillage, the average aggregate-associated C contents of the large and small macroaggregates and microaggregates under T1, T2, and T3 were 11.60 g·kg^−1^, 12.14 g·kg^−1^ and 12.33 g·kg^−1^, respectively. These values under organic fertilizer and straw return were significantly higher than those under single chemical fertilizer. Similar results were obtained under the other two tillage modes.

### Crop yield

**Figure 1:**
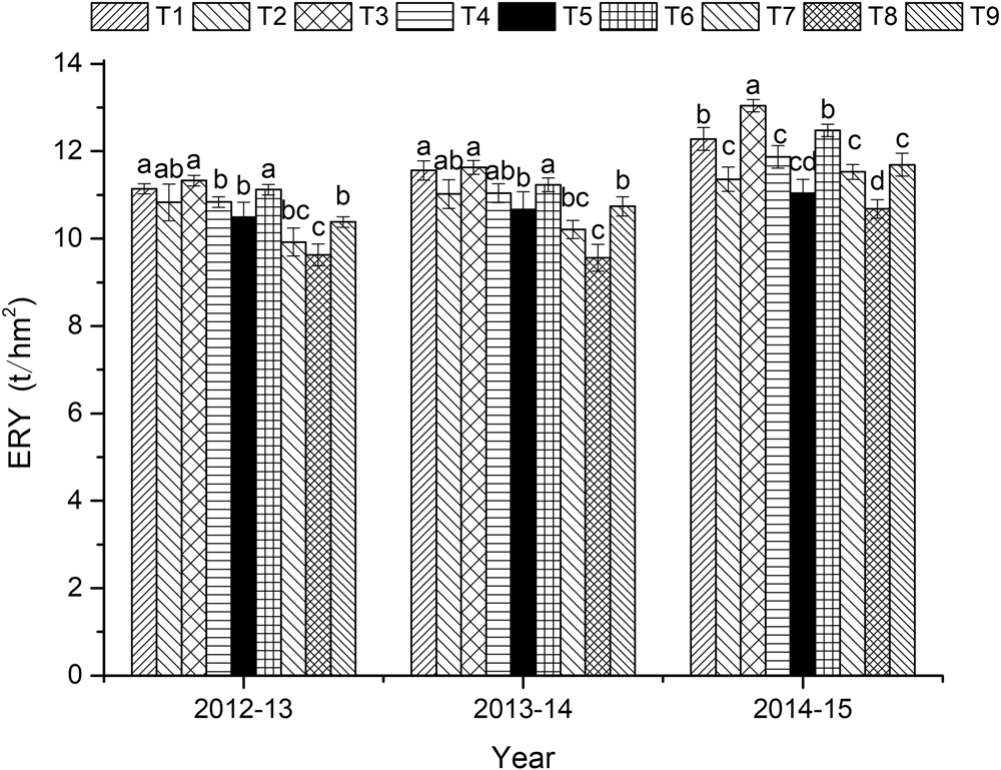
Equivalent rice yield (ERY) under different treatments in the year 2012–13, 2013–14 and 2014–15. The different lowercase letters above the bars in the same year indicate significant differences at *P* < *0*.*05*. Treatment abbreviations are as listed in Table 1.

As shown in Fig. 1, the rice yield in 2015 was generally higher than that in the previous 2 years. However, no-tillage and straw return decreased the rice yield. Conventional tillage with chemical fertilizer (T1) produced a yield 6.50% higher than that under no-tillage coupled with chemical fertilizer (T7). Under the same tillage, the rice yield under straw return was lower than that under the other two treatments that had no straw return. This trend became more pronounced in each consecutive planting year. Straw return decreased yield 14.78% compared to that under organic fertilizer, this difference was significant in 2015. The rice yield was the highest under conventional tillage coupled with organic fertilizer (T3), which increased yield by 22.10% compared to that under no-tillage coupled with straw return (T8) in the same season.

### Crop yield components

The above yield results are attributed mainly to the effects of the different tillage, straw return and fertilizer treatments on crop yield components. No-tillage and straw return had negative effects on rice growth and development. As shown in Table 4, under the same fertilizer conditions, rice plant height under conventional tillage (T1) and rotary tillage (T4) was 11.23% and 10.00% higher, respectively, than that under no-tillage (T7) 14 days after transplanting and 4.11% and 3.66% higher, respectively, at harvest. Under the same conventional tillage, straw return (T2) also decreased rice plant height compared to that under T1 and T3 14 days after transplanting. The same results were observed under rotary tillage and no-tillage. In addition to the effects on the seedlings, no-tillage also reduced the effective panicle number of rice at harvest. As shown in Table 5, the effective panicle number of rice under conventional tillage and rotary tillage was higher than that under no-tillage. No-tillage coupled with straw return (T8) produced a 10.92% lower effective panicle number than that under conventional tillage coupled with organic fertilizer (T3), which produced the highest value of all treatments. Therefore, the difference in the effective panicle number was the main factor impacting rice yield.

**Table 4:** Effective panicle number (EP), grain number per panicle (GNP), 1000-grain weight (1000-GW) and seed setting rate (SSR) of rice and wheat under different treatments in the planting year 2014–2015.

Treatments	Rice				Wheat			
	EP	GNP	1000-GW	SSR	EP	GNP	1000-GW	SSR
	(×10^4^·hm^−2^)		(g)	(%)	(×10^4^·hm^−2^)		(g)	(%)
T1	332.60 ± 8.26a	120.82 ± 3.24a	27.42 ± 0.14a	94.23 ± 0.21a	665.12 ± 15.68a	35.12 ± 0.24a	41.22 ± 0.54a	94.82 ± 1.56a
T2	311.22 ± 12.47b	119.48 ± 2.10a	27.90 ± 1.37a	93.54 ± 0.54a	602.18 ± 13.24ab	33.20 ± 1.56b	41.38 ± 0.32a	92.56 ± 1.24a
T3	335.51 ± 2.38a	120.37 ± 2.28a	27.22 ± 0.65a	93.64 ± 0.37a	712.34 ± 14.82a	32.36 ± 0.58b	41.53 ± 0.42a	93.68 ± 0.89a
T4	328.43 ± 4.82a	120.80 ± 5.13a	27.71 ± 0.38a	94.72 ± 0.16a	639.61 ± 9.84b	32.38 ± 2.14b	41.78 ± 0.28a	91.79 ± 1.28a
T5	310.25 ± 7.44b	119.22 ± 1.04a	27.90 ± 0.82a	93.88 ± 0.24a	608.62 ± 12.04ab	32.44 ± 0.35b	40.35 ± 0.78a	92.25 ± 0.65a
T6	334.24 ± 7.26a	120.31 ± 2.37a	27.15 ± 1.10a	94.21 ± 0.28a	687.52 ± 8.65a	35.24 ± 0.37a	41.21 ± 0.21a	94.84 ± 2.31a
T7	318.27 ± 1.54b	119.34 ± 1.05a	27.59 ± 0.68a	93.66 ± 0.87a	589.37 ± 12.14bc	30.52 ± 1.55b	40.01 ± 1.23a	90.62 ± 1.14a
T8	302.49 ± 7.10c	119.52 ± 2.05a	27.77 ± 0.49a	93.92 ± 0.92a	567.52 ± 10.85c	30.22 ± 1.24b	40.51 ± 0.95a	89.87 ± 1.20a
T9	310.46 ± 6.85b	119.22 ± 2.26a	27.51 ± 1.11a	93.45 ± 0.21a	564.24 ± 16.54c	30.14 ± 1.12b	40.74 ± 1.24a	88.86 ± 1.15a

Mean values are shown ± SEs (n = 3), and different lowercase letters in the same column indicate significant differences at *P* < *0*.*05*. Treatment abbreviations are as listed in Table 1.

**Table 5:** Tillage, straw return and fertilization treatments.

Treatments	Cultivation management	Abbreviations
T1	Conventional tillage coupled with chemical fertilizer	CC
T2	Conventional tillage coupled with straw return and chemical fertilizer	CSC
T3	Conventional tillage coupled with organic fertilizer and chemical fertilizer	COC
T4	Rotary tillage coupled with chemical fertilizer	RC
T5	Rotary tillage coupled with straw return and chemical fertilizer	RSC
T6	Rotary tillage coupled with organic fertilizer and chemical fertilizer	ROC
T7	No-tillage coupled with chemical fertilizer	NC
T8	No-tillage coupled with straw return and chemical fertilizer	NSC
T9	No-tillage coupled with organic fertilizer and chemical fertilizer	NOC

As in rice, no-tillage and straw return decreased the effective panicle number of wheat. The mean effective panicle numbers under the three conventional tillage treatments (T1, T2, and T3) and under the three rotary tillage treatments (T4, T5, and T6) were 15.02% and 12.47% higher, respectively, than those under the three no-tillage treatments (T7, T8, and T9). In addition to tillage, straw return coupled with conventional tillage (T2) decreased the effective panicle number by 18.29% and 10.45% compared to that under organic fertilizer (T3) and single application of chemical fertilizer (T1) with conventional tillage.

## Discussion

**Figure 2:**
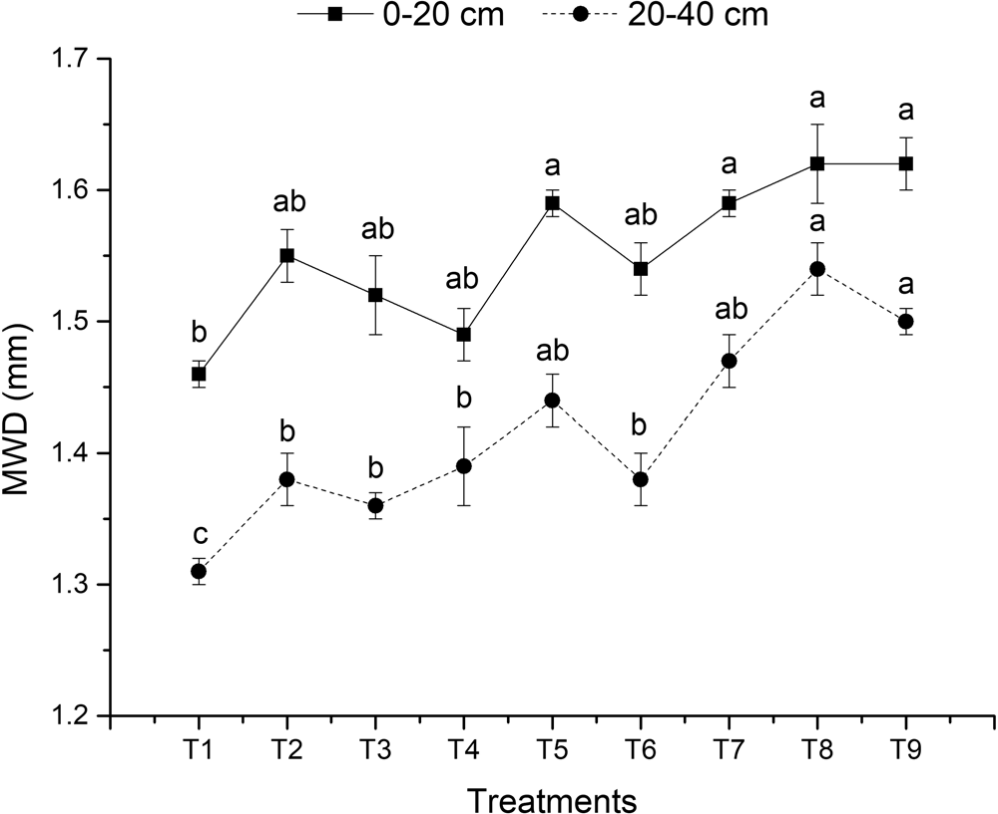
The mean weighted diameter (MWD) of the soil aggregates in topsoil and subsoil under different treatments after 3 years of rice-wheat cropping rotation. The different lowercase letters above the squares or dots in the same soil layer indicate significant differences at *P* < *0*.*05*. Treatment abbreviations are as listed in Table 1.

Different tillage, straw return and fertilization treatments all had an impact on the content and stability of soil aggregates. Among them, the tillage had the most important impact on soil aggregates. Compared to conventional tillage, no-tillage caused large macroaggregates, small macroaggregates and aggregates to increase by 50.07%, 26.33% and 14.38%, respectively. The decrease in macroaggregates in conventional tillage might be due to the destruction of large particles, resulting in the oxidation of previously protected SOC^26^. Six *et al*.^27^ proposed that mechanical disturbances reduce the soil structure stability, especially the structure of macroaggregates, of various soil types and under various conditions. Our results also showed that the mean weighted diameter (MWD) of soil aggregates in no-tillage was significantly higher than that of conventional tillage (Fig. 2). Because the alluvial soil in the study area was formed by sediment deposition, it is neutral loam and the stability of the soil structure is poor^28^. It is easily broken down by the destruction of external mechanical forces. Macroaggregates were more susceptible to damage than microaggregates. The reason is that microaggregates are formed mainly by short range van-der-Waals forces and electrostatic binding (including ions, predominantly cations) between soil particles^29,30^. However, macroaggregates mainly form by microaggregates combining with binders (e.g. roots and hyphae)^31^. Therefore, macroaggregates are not as stable as microaggregates, and their turnover rate in soil is faster and more susceptible to mechanical tillage.

In addition to tillage, straw return was also an important factor in promoting the formation of soil aggregates, as management of crop residues is the key to soil structure development and stability^32^. The significant increase of SOC and macroaggregates we observed in no-tillage could mainly be due to the combination of crop residues (shallow roots and stubble) and the decomposition of straw residues on the soil surface. This releases polysaccharides and organic acids, which play major roles in stabilizing aggregates^33^. They combined with soil particles *in situ* to form aggregates^34^. The newly added residues could also serve as nucleation sites for fungal and other soil microbial growth^35^, all of which increased water-stable aggregates. Therefore, the combination of no-tillage and straw return improved the formation of water-stable aggregates and also led to a higher proportion of macroaggregates in the soil^36^

Many studies have reported that SOC content was lower in conventional tillage than no-till^37–39^. Our results also showed that no-till presented an increase in TC, SOC and LOC of 28.87%, 28.50% and 37.11%, respectively, compared to conventional tillage. The destructive influence of tillage lead to loss of SOC by increasing soil microbial respiration^40^. No-tillage limited soil disturbance and enhanced the physical protection of C by aggregates. Al-Kaisi’s^41^ study has shown that the correlation between the percentage of SOC content of all tillage systems and the content and stability of macroaggregates and microaggregates was positive (r = 0.65), and NT showed the highest percentage of SOC content. The significant increase we observed in macroaggregates and SOC with straw return, compared to single application of chemical fertilizers, was mainly due to the combination of crop residues and soil. The straw residue on the soil surface could be mineralized and stabilized as soil organic matter^42^. Macroaggregates are usually formed by the combination of organic residues and microaggregates^43^. Macroaggregates are very susceptible to oxidation^44^, but no-tillage and straw return can increase the content of macroaggregates, and also provides protection for SOC^45^. Regulating proper ventilation and water infiltration in the root zone was shown to result in higher proportions of macroaggregates, ensuring more carbon sequestration and nutrient availability^46^. Mikha and Rice^47^ found that aggregates with diameters between 2.0–0.25 mm were the main carriers of organic carbon. This was consistent with our results. More SOC was fixed because medium-sized aggregates, contain more active sites due to their higher specific surface area^48^. Therefore, they are capable of adsorbing organic substances through stronger ligand exchange and multivalent cation bridges^49^. Consequently, their aggregate associate-C is even higher than that of the large macroaggregates and microaggregates.

In our study, the trend of SOC content under different treatments in the subsoil was different from that in the topsoil. In the topsoil, no-tillage showed higher SOC than that of conventional tillage. However, the subsoil content of SOC under conventional tillage was higher than that under no-tillage, with conventional tillage >rotary tillage >no-tillage. This could be because the depth of conventional tillage can reach 30–40 cm so the crop straw covering the soil surface and organic matter such as roots and organic fertilizers remaining in the shallow soil would be moved into deeper soil layers. These organic substances were tightly bound to soil particles, and the mineralization stability was higher, which promoted the accumulation of organic carbon in deep soils^50^. Another possible mechanism was that tillage carries the organic matter and nutrients of the soil surface into the subsoil, providing more C source and growth nucleation sites for fungi and soil microorganisms, promoting the formation of aggregate associated-C^51^.

**Figure 3:**
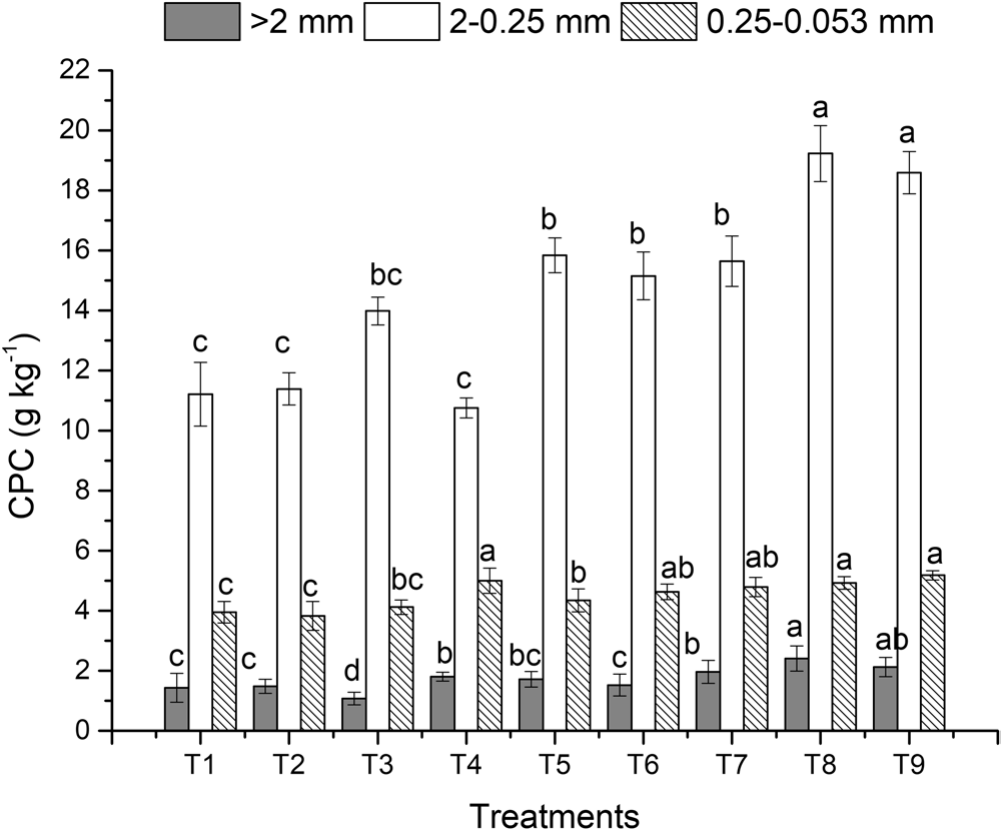
The organic carbon preservation capacity (CPC) of the soil aggregates in topsoil under different treatments after 3 years of rice-wheat cropping rotation. The different lowercase letters above the bars for the same aggregates indicate significant differences at *P* < *0*.*05*. Treatment abbreviations are as listed in Table 1.

**Figure 4:**
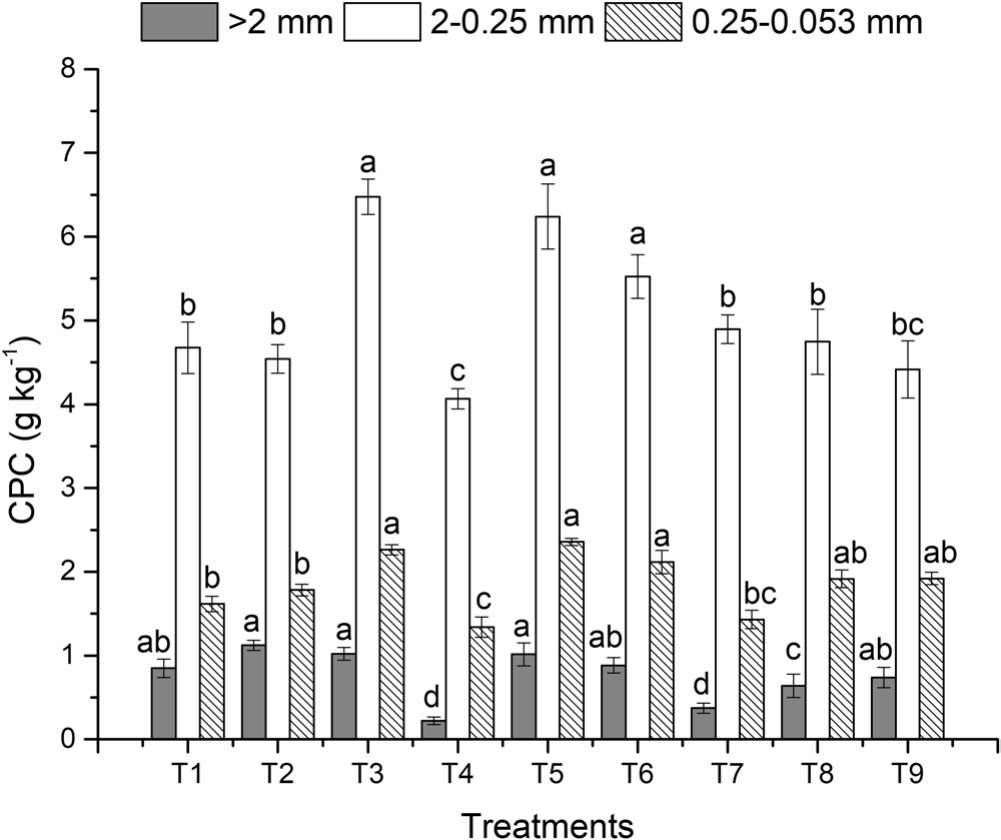
The organic carbon preservation capacity (CPC) of the soil aggregates in subsoil under different treatments after 3 years of rice-wheat cropping rotation. The different lowercase letters above the bars for the same aggregates indicate significant differences at *P* < *0*.*05*. Treatment abbreviations are as listed in Table 1.

The analysis of the CPC also corroborated that no-tillage and straw return increased the carbon storage capacity of the aggregates. Of the different size-aggregates, small macroaggregates presented the highest C capture capacity, followed by microaggregates, then large macroaggregates. Higher C density in small macroaggregates indicated their role in C sequestration in soil. Tillage, straw return and fertilization affected the CPC of the various aggregate fractions. In the topsoil (Fig. 3), no-tillage had higher CPC than conventional and rotary tillage. Under the same tillage, straw return and applying organic fertilizer could increase aggregate-associated C. This could due to the fact that no-tillage preserved the porous structure of the macroaggregates, and straw return or organic fertilizer directly contributed to C enrichment^43^. In contrast with the topsoil, conventional tillage had higher CPC in the subsoil than no-tillage (Fig. 4), which indicated that the input of organic carbon became the determining factor of aggregate-associated C as the soil layer deepened and soil interference decreased. Compared to no-tillage, conventional tillage added more organic matter to the subsoil, thus enhancing CPC in the aggregates. This also indicated that the subsoil had a great carbon sequestration potential. Therefore, processes that increase organic matter content in the subsoil are more important for raising CPC in the soil profile.

There is a major controversy whether conservation tillage can increase crop yields. Some studies suggested that conservation tillage could continuously improve soil structure, building better crop growing conditions, increasing SOC content, and increasing crop yield^26,52,53^. However, other studies have shown that conservation tillage increased aggregates and organic carbon in soil surface, but reduced crop yields^54,55^. The results of this study showed that crop yield in conservation tillage was lower than that of conventional tillage, and the difference became more pronounced with time. By the third year conservation tillage resulted in a 10.63% and 7.82% reduction in rice and wheat yield, respectively, compared with conventional tillage. Analysis of the yield components of rice and wheat indicated that conservation tillage retarded the growth of rice and wheat seedlings. Compared with conventional tillage, no-tillage coupled with straw return resulted in a decrease of 11.23% in rice and 10.00% in wheat plant height, respectively 14 days after sowing. This effect also led to a decrease in the effective panicle number (EP) of rice and wheat when harvested. Compared with conventional tillage, no-tillage and straw return decreased the EP of rice and wheat by 10.92% and 10.45%, respectively. There could be three main reasons for this result. Firstly, no-tillage reduced the permeability of air and water to the rhizosphere. Reducing tillage often leads to surface sealing, which reduces water infiltration and affects seedling root development^55^. The studies by Prasad^54^ in semi-arid tropical regions of India showed that rice growth and yield decreased with reduction of tillage. This was because as the intensity of tillage decreased, rainfall had difficult infiltrating the soil, and soil moisture became the main factor restricting crop yield. Secondly, during the 3 years of this study, all rice and wheat straw was returned to the field and a large amount of straw did not degrade on the soil surface of no-tillage treatments. This hindered the penetration of crop seeds into the soil and slowed seedling growth. Thirdly, the C/N increased in the soil after straw return. This was probably because soil microorganisms needed to assimilate nitrogen in order to make the enzymes needed to degrade straw. They therefore competed for N with crop seedlings, which would also reduce seedling growth and development. Derpsch *et al*.^56^ also suggested that no-till reduced the effectiveness of nitrogen fertilization compared to conventional tillage, resulting in a significant reduction in wheat yield (29%). Therefore, the results of this study indicated that reducing the intensity of tillage had a significant negative impact on crop yield. This was also the main factor impeding the large-scale promotion of conservation tillage in the study area. It is necessary to propose effective solutions both to raise SOC and to maintain crop yields. One method is to improve the soil moisture content during the sowing period of crop, especially for wheat. Another method is to enhance the nitrogen application to lower the C/N. The third is to find an effective method for rapid degradation of crop straw. This might be done by crushing the straw into smaller pieces, applying straw decomposing agents or inoculating earthworms to degrade straw and improve air and water permeability in the topsoil^57^.

The soil of Chongming Island is a sandy soil with light texture, poor adhesion, and high salt content but low organic matter and nutrient contents. A rice-and-wheat rotation system has long been practiced in this region, with two crops per year and a high multiple cropping index. Moreover, since straw can be burned as fuel, it is removed from farmland, with no straw return. This practice, coupled with the scarcity of organic fertilizer, which has led the SOC content to decrease over time. In recent years, to improve the quality of cultivated land and increase the SOC content, the local government has vigorously promoted measures of no-tillage, less tillage and full return of rice and wheat straw. In the absence of organic fertilizer, the practice of straw return to the field is indeed an effective measure to increase the SOC content. On one hand, straw itself is a source of organic carbon and can increase organic carbon input. On the other hand, due to the texture of estuary alluvial soil being sand-prone with poor adhesion, the practice of no-tillage improves soil structure stability. Moreover, the straw itself contains binders such as polysaccharides that are beneficial to the formation and stability of soil aggregates, physically protecting the carbon. However, because no-tillage and straw return have been practiced only for a short time, and the land capacity has not yet been effectively improved, which has resulted in decreased rice and wheat yields. These measures have not been widely accepted by local farmers and thus are being implemented rather slowly. The results of this study indicate that no-tillage and straw return are indeed beneficial to the formation and stability of soil aggregates and promote the absorption of organic carbon, while no-tillage is more conducive to the fixation of SOC in surface soil, and plowing in combination with straw return is more conducive to the accumulation of organic carbon in subsurface soil. However, compared to conventional tillage, no-tillage and straw return sustained less crop yield.

## Conclusion

Our results suggest that no-tillage and straw return in rice-wheat cropping rotation systems is an effective management practice for the formation and stability of soil aggregates, even in the estuarine alluvial soil of Chongming Island. This practice has shown potential to increase the number of water-stable macroaggregates and microaggregates in both the topsoil and subsoil compared to that under conventional tillage. The results of this study showed that straw return and application of organic fertilizer increased the cumulative carbon input and increased the aggregate-associated C content. Moreover, straw return is a better option for improving CPC than application of organic fertilizer in rice-wheat cropping rotations. Higher SOC stocks were observed in the subsoil under straw return with conventional tillage than under straw return with no-tillage. This suggests that the benefits of no-tillage in sequestering SOC are concentrated mostly to the topsoil, while conventional tillage is more conducive to SOC accumulation in the subsoil. We also found that no-tillage and straw return showed lower rice and wheat yields. The key reason for this result is that the negative effect on crop seedlings resulted in decreased EP in rice and wheat. As a consequence, straw return was able to increase SOC stocks under both no-tillage and conventional tillage. However, the overall potential of no-tillage to enhance SOC stocks and improve crop yields is very likely to be overestimated, which suggests that conventional tillage coupled with straw return is a possible option with multiple advantages in estuarine alluvial soil.

## Methods

### Study area description

This investigation was conducted at Sanxing Experimental Station (SES) on Chongming Island (31°41′20″N, 121°33′47″E, altitude ranging from 3.5–4.5 m), China, with a rice (*Oryza sativa* L.) – wheat (*Triticum aestivum* L.) cropping rotation system. Chongming Island, 1267 km^2^ in area, is an alluvial island in the Yangtze River Delta with a flat terrain and a land formation time of approximately 1300 years. It is in the north subtropical zone and has a typical subtropical monsoon climate, with an annual average temperature of 15.3°C, an annual average precipitation of 1003.7 mm, an annual average of 2104 sunshine hours, and an approximately 229 day frost-free period. The soil in the experimental field was estuarine alluvial soil, with 15.21 g·kg^−1^ soil organic matter, 0.94 g·kg^−1^ total N, 81.62 mg·kg^−1^ alkali-hydrolysable N, 53.53 mg·kg^−1^ available P, 109.35 mg·kg^−1^ available K, and pH 8.30 (soil-water ratio 5:1).

### Test materials

The rice cultivar in this study was Han you 8, its seeding rate was 187.5 kg·hm^−2^, and the row spacing was 23 cm. The wheat cultivar was Wanmai 52, its seeding rate was 120 kg·hm^−2^, and the row spacing was 25 cm. The straw returned in the wheat season was the rice straw harvested from the previous season (C/N = 53.11, in which the contents of cellulose, hemicellulose, and lignin were 36.43%, 22.52%, and 18.69%, respectively). The straw returned in the rice season was the wheat straw harvested from the previous season (C/N = 81.72, in which the contents of cellulose, hemicellulose, and lignin were 33.62%, 22.53%, and 18.45%, respectively). N, P and K were supplied through urea (N-46%), calcium superphosphate (P_2_O_5_–46%), and potassium chloride (K_2_O-60%). Organic fertilizer was decomposed pig manure. The contents of N, P, and K in the organic fertilizer were 10.52 mg·kg^−1^ N, 20.47 mg·kg^−1^ P_2_O_5_, and 15.13 mg·kg^−1^ K_2_O, respectively.

### Experimental design and field management

The experiment began in October 2012 and included a total of nine treatments combining different tillage systems, fertilization types, and the presence/absence of straw return (Table 1). Each treatment included three replications and was set up in a single-factor random block design. Each plot was 40 m long and 6 m wide, with an area of 240 m^2^. The ridges between the plots and the irrigation/drainage ditch ridges between the blocks were separated by plastic film.

The depth of the conventional tillage was 30 cm, and the depth of the rotary tillage was 20 cm. In the straw return plots, all rice and wheat stalk biomass (including straw, root and stubble) was returned to the soil under T2, T5, and T8 treatments. (Crop straw was crushed into 8–10-cm-long segments and distributed on the field of each plot by a fully automatic harvester). In the plots without straw return, the aboveground portion of the crops were removed from the plot during crop harvesting (only roots and very little stubble remained in the field). The chemical fertilizer application rates in the rice season were 225 kg·hm^−2^ N, 90 kg·hm^−2^ P_2_O_5_, and 90 kg·hm^−2^ K_2_O, and the application rates in the wheat season were 270 kg·hm^−2^ N, 60 kg·hm^−2^ P_2_O_5_, and 90 kg·hm^−2^ K_2_O. The application rate of organic fertilizer was 12,500 kg·hm^−2^ in both the rice and wheat seasons. Straw, organic fertilizer, P fertilizer, and K fertilizer as base fertilizers were applied once before planting in each crop season. The N fertilizer included two parts, base fertilizer and topdressing. To each plot, 60% of the N and all of the P, K and organic fertilizers were applied before sowing in the rice season. The remaining N was applied in two equal parts 20 and 60 days after sowing. In the wheat season, 60% of the N and all of the P, K and organic fertilizers were applied before sowing. The remaining N was applied in equal amounts 10, 30 and 60 days after sowing. Field management and pest control were performed according to the local management routines.

### Sample collection and analysis

The soil samples were collected in September 2012 before the start of the experiment. During the experiment, soil samples were collected in the topsoil (0–20 cm) and subsoil (20–40 cm) by a 5-cm-diameter corer in each plot after the wheat harvest (in June 2013, June 2014, and June 2015) and after the rice harvest (in November 2013, November 2014, and November 2015). The collected soil samples were transported to the laboratory in a timely manner, and animal and plant debris and stones were removed. The soil samples were air-dried in the shade. A portion of the soil samples was ground and passed through a 0.149-mm sieve. The parameters that were measured included soil TC, inorganic carbon, organic carbon, active organic carbon, and nutrients. The other portion of the air-dried soil samples was used to determine the soil aggregate composition. The soil aggregate composition was measured by the wet-sieving method, in which the soil aggregates of different particle sizes were obtained by using a soil aggregate analyzer (DIK-2001) and included large macroaggregates (>2.0 mm), small macroaggregates (2–0.25 mm), and microaggregates (0.25–0.053 mm)^58^.

In addition, the TC content was measured by a CHN-440 element analyzer. The SOC content was determined by the external-heat K dichromate oxidation method. The LOC content was determined by the diluted-heat K dichromate oxidation method^59^.

### Data calculation and analysis

The mass percentage of aggregates at a given particle size level was calculated as^39^.

$$(Ai \% )=MAi/Ms\ast 100 \% $$

where MA_i_ is the mass of the aggregates of a given particle size level (>2.0 mm, 2–0.25 mm, or 0.25–0.053 mm), and Ms is the mass of the air-dried soil sample.

The MWD of the soil aggregates was calculated as^39^.

$$(MWD)={\sum }_{i=1}^{n}DiMAi/{\sum }_{i=1}^{n}MAi$$

where n = 3 is the number of the particle size levels of soil aggregates (>2.0 mm, 2–0.25 mm, or 0.25–0.053 mm), D_i_ is the mean diameter of the aggregates of each particle size level (2.0 mm, 1.125 mm, and 0.152 mm), and MA_i_ is the mass of aggregates of the corresponding particle size level.

The organic CPC of the soil aggregates was calculated as^39^.

$$(CPC)=\frac{MACi\ast MAi}{100},$$

where MACi is the organic carbon content of the aggregates of a given particle size level (>2.0 mm, 2–0.25 mm, or 0.25–0.053 mm), and MAi is the content of aggregates of a given particle size level (>2.0 mm, 2–0.25 mm, or 0.25–0.053 mm).

The rice yield was calculated as^39^

$$(ERY)=RY+\frac{WY\ast WP}{RP}$$

where RY is the rice yield, WY is the wheat yield, RP is the governmental purchase price of rice in that year, and WP is the governmental purchase price of wheat in that year.

The data analysis focused mainly on the soil samples collected in November 2015 (3 years after the experiment began). Data were first sorted with Microsoft Excel 2010, and analysis of variance (ANOVA) was then conducted with the software package SPSS 17.0, in which the Tukey method was used to perform multiple comparisons among the different treatments. Graphs and figures were created with Origin 8.0.
